# Living in the Southern Hemisphere: Metabolic Syndrome and Its Components in Amazonian Riverine Populations

**DOI:** 10.3390/jcm10163630

**Published:** 2021-08-17

**Authors:** Gabriela P. Arrifano, Jacqueline I. Alvarez-Leite, Barbarella M. Macchi, Núbia F. S. S. Campos, Marcus Augusto-Oliveira, Letícia Santos-Sacramento, Amanda Lopes-Araújo, José Rogério Souza-Monteiro, Raquel Alburquerque-Santos, José Luiz M. do Nascimento, Sidney Santos, Ândrea Ribeiro-dos-Santos, Reinaldo B. Oriá, Maria Elena Crespo-Lopez

**Affiliations:** 1 Laboratório de Farmacologia Molecular, Instituto de Ciências Biológicas, Universidade Federal do Pará, Belém 66075-110, Brazil; arrifanogabriela@gmail.com (G.P.A.); nfernanda@ufpa.br (N.F.S.S.C.); marcusoliveira@globo.com (M.A.-O.); letisacramentolfm@gmail.com (L.S.-S.); amanda.lopes1647@gmail.com (A.L.-A.); raquel32abq@gmail.com (R.A.-S.); 2 Laboratório de Aterosclerose e Bioquímica Nutricional, Departamento de Bioquímica e Imunologia, Universidade Federal de Minas Gerais, Belo Horizonte 30161-970, Brazil; jalvarezleite@yahoo.com.br; 3 Laboratório de Neuroquímica e Biologia Celular, Instituto de Ciências Biológicas, Universidade Federal do Pará, Belém 66075-110, Brazil; bmacchi@gmail.com (B.M.M.); jlmn@ufpa.br (J.L.M.d.N.); 4 Faculdade de Medicina, Campus de Altamira, Universidade Federal do Pará, Altamira 68372-040, Brazil; rogerio.souza.monteiro@gmail.com; 5 Programa de Pós-Graduação em Ciências Farmacêuticas, Departamento de Ciências Biológicas e da Saúde, Universidade Federal do Amapá, Macapá 68903-419, Brazil; 6 Laboratório de Genética Humana e Médica, Instituto de Ciências Biológicas, Universidade Federal do Pará, Belém 66075-110, Brazil; sidneysantos@ufpa.br (S.S.); akely@ufpa.br (Â.R.-d.-S.); 7 Laboratório de Biologia da Cicatrização, Ontogenia e Nutrição de Tecidos, Faculdade de Medicina, Universidade Federal do Ceará, Fortaleza 60430-160, Brazil; oria@ufc.br

**Keywords:** cardiovascular disease, cholesterol, hypertension, risk factor, dyslipidemia, HDL, nutritional, diabetes

## Abstract

The metabolic syndrome (MetS) epidemic is a global challenge. Although developing countries (including Brazil, India, and South Africa) present a higher proportion of deaths by cardiovascular diseases than developed countries, most of our knowledge is from these developed countries. Amazonian riverine populations (ARP), as well as other vulnerable populations of the Southern Hemisphere, share low-income and traditional practices, among other features. This large cross-sectional study of ARP (*n* = 818) shows high prevalence of hypertension (51%) and obesity (23%). MetS was diagnosed in 38% of participants (especially in women and 60–69 years-old individuals) without the influence of ancestry. Only 7–8% of adults had no cardio-metabolic abnormalities related to MetS. Atherogenic dyslipidemia (low HDL-cholesterol) was generally observed, including in individuals without MetS. Still, slight differences were detected between settings with a clear predominance of hypertension in Tucuruí. Hypotheses on possible genetic influence and factors (nutrition transition and environmental pollutants -mercury) are proposed for future studies. Moreover, a roadmap to MetS progression based on the most prevalent components is provided for the development of tailored interventions in the Amazon (initially, individuals would present low HDL-cholesterol levels, later progressing to increased blood pressure characterizing hypertension, and ultimately reaching MetS with obesity). Our alarming results support the need to improve our knowledge on these vulnerable populations.

## 1. Introduction

The metabolic syndrome (MetS) epidemic is a global challenge affecting approximately 25% of the adult population worldwide [1]. MetS is a clustering of metabolic, biochemical, and clinical abnormalities, characterized by elevated waist circumference, increased fasting blood glucose (FBG), high blood pressure, elevated triglycerides, and/or low high-density lipoprotein-cholesterol (HDL-c) in the blood (i.e., atherogenic dyslipidemia) [1,2]. The clinical diagnosis of MetS is essential for the early identification of patients at risk of developing cardiovascular diseases (CVD), type 2 diabetes mellitus (T2DM), atherosclerosis, nonalcoholic fatty liver disease, polycystic ovary syndrome, and dementia [3,4,5]. MetS increases overall mortality by 1.5-fold, the risk of T2DM by 5-fold, and the risk of developing CVD over the next 5–10 years by 2-fold [2]. Currently, CVD are the leading causes of death at the global, regional, and country-specific levels [6]. All members of the World Health Organization (WHO) committed to reducing premature deaths originating from non-communicable diseases (NCD) by 25% in 2025, including CVD [7]. The first step is the early identification of individuals at risk. Then, both patients and clinicians can decide whether lifestyle modification and preventive medical treatment are required to avoid the disease’s establishment. 

Although developing countries, such as Brazil, India, and South Africa, present higher proportions of CVD deaths in the working age as compared to the USA and Portugal [8,9,10], most of our knowledge regarding MetS epidemiology is from developed countries where urbanization, sedentary lifestyle, and access to ultra-processed and fast foods contribute to this pandemic syndrome. In underdeveloped/developing countries, our knowledge is especially scarce with vulnerable populations such as those of the Amazon region, where, in addition to the miscegenation between African, European, and indigenous ancestries [11], these populations face very different challenges of those found in developed countries. The definition of vulnerable populations is broad and include pregnant women/infant/children, but also, all those individuals in economic disadvantage, racial and ethnic minorities, among others, including rural populations who frequently encounter barriers to accessing healthcare services [12,13]. Amazonian riverine populations share with other vulnerable populations of the Southern Hemisphere features such as rural and remote locations far from city centers, limited access to healthcare facilities, poor sanitary conditions, traditional practices, and low-income, among others [11,14,15,16]. In Amazonian riverine populations, the river is central to life, and they usually present a subsistence economy mainly based on fishing and familiar agriculture (fruits, vegetables, manioc flour, etc.) [17]. In theory, the riverine lifestyle should be relatively healthier (surrounding by nature, less stressful life, fish as the main protein of the diet, etc.) than that of the urban dweller. However, recent data of riverine populations showed a significantly higher prevalence of NCD such as systemic arterial hypertension and impaired glucose tolerance or *Diabetes Mellitus*-suspected status as compared to urban populations [15] supporting the need for a better understanding of the epidemiology of MetS. 

Thus, the present work investigated the components and prevalence of MetS in Amazonian riverine populations, providing insights and future directions for research based on the nutritional status, hypertension, lipid profile, and prevalence of MetS components.

## 2. Materials and Methods

### 2.1. Ethical Aspects and Populations

The National Council for Ethics in Research with Humans approved this work (CONEP, Brazil; CAAE nº 43927115.4.0000.0018). The recruitment of participants was performed in 2015–2018 by announcing the project via radio, meetings, and direct communications with healthcare agents. Samples were collected from volunteer participants at community meeting places such as schools. After a detailed explanation about the study, informed consent was obtained from all participants. This study followed the ethical principles of the Declaration of Helsinki for human research and it is in accordance with the STROBE guidelines [18]. 

**Figure 1:**
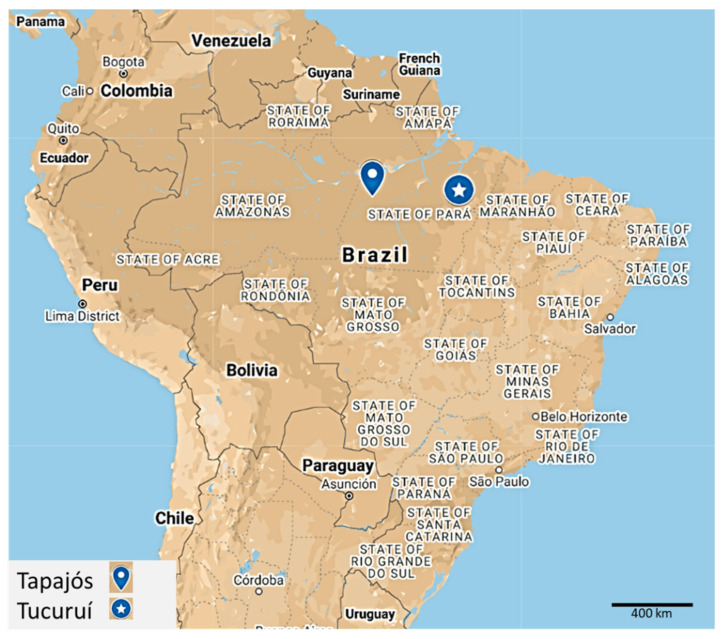
Map of the States of Brazil with the approximate locations of the two regions where individuals were enrolled for this study: The Tapajós River basin and the Tucuruí Lake. This map was designed using MyMaps of Google and it can be consulted at: https://www.google.com/maps/d/u/0/edit?mid=1u0_9V0_jI_RYzJ1Ed1n2UM1apLe4tH7Z&usp=sharing (accessed on 1 August 2021).

The inclusion criteria were adult volunteers (≥18 years old) of riverine populations (living in the community for at least the last two years) of two Amazonian regions (Figure 1): Tapajós Basin (−4.287121, −55.984106) and Tucuruí Lake (−3.800897, −49.811848). The exclusion criteria included participants without venous access or not enough serum or who refused to donate blood.

### 2.2. Data and Sample Collection 

Anthropometric data (height, weight, and waist circumference) were registered without shoes, coats, jackets, caps, and any other accessory that could influence the final measurements, according to [17]. Waist circumference was measured using a non-elastic tape 2 m long, with a precision of 0.1 cm, positioned at the horizontal plane midway between the lowest ribs and the iliac crest [19]. Body mass index (BMI) was calculated by the equation weight (kg)/height (m)^2^. BMI was used to indicate nutritional status according to WHO [20]: Underweight (<18.5 kg/m^2^), normal weight (18.5–24.9 kg/m^2^), overweight (25.0–29.9 kg/m^2^), and obesity (≥30.0 kg/m^2^). Means of two different evaluations of blood pressure after ten minutes rest were registered as previously described [15]. After an overnight fast, blood was collected and processed for serum separation. 

### 2.3. Biochemical Analysis and Definition of MetS

FBG, total cholesterol, triglycerides, and HDL-c were analyzed using commercial kits (Labtest Diagnostica, Lagoa Santa, Brazil). Non-HDL-c was calculated as total cholesterol minus HDL-c. MetS was defined according to the American Heart Association [21], in those presenting three or more of the following alterations: Systolic blood pressure (SBP) ≥130 mm Hg and/or diastolic blood pressure (DBP) ≥85 mm Hg, FBG ≥ 100 mg/dL, triglyceridemia ≥ 150 mg/dL, HDL-c < 40 mg/dL for men and <50 mg/dL for women, and/or waist circumference > 102 cm and >88 cm for men and women, respectively. Alternatively, BMI ≥ 30.0 kg/m^2^ was used in MetS diagnosis to replace missing waist circumference data [22]. 

### 2.4. Ancestry

Genomic ancestry analysis was performed based on the method previously described [11], assuming three parental populations (European, African, and Amerindian) and using 61 autosomal ancestry informative markers. Two multiplex PCR reactions of 20 and 22 markers were performed and amplicons were analyzed by electrophoresis using the ABI Prism 3130 sequencer (Applied Biosystem, Foster City, CA, USA) and GeneMapper ID v.3.2 software (Applied Biosystem, Foster City, CA, USA). The individual proportions of European, African, and Amerindian genetic ancestries were estimated using STRUCTURE v.2.3.3 software (Pritchard Lab, Stanford University, Stanford, CA, USA).

### 2.5. Statistical Analysis

According to the results of the Kolmogorov–Smirnov normality test, differences between two groups were analyzed with the Mann–Whitney U test. Proportions were compared using the Chi-square or Fisher’s exact tests when appropriate. For all analysis, *p* < 0.05 was considered significant.

## 3. Results

A total of 883 riverine participants were enrolled in the present study, and complete data for the analysis of MetS were available for 818 participants, 417 from the Tapájos River basin and 401 from the Tucuruí Lake region (Table 1). Noteworthily, despite the absence of complete data for 65 participants (due to the absence of venous access or not enough serum, duplicate registration, or refusal to donate blood), 15 of the excluded participants (23%) already met the criteria for MetS diagnosis (i.e., they showed at least three altered components).

Overall, riverine participants show overweight (>25 kg/m^2^), low HDL-c levels (<40 mg/dL), high non-HDL cholesterol (>130 mg/dL), and borderline cholesterol (>190 mg/dL) [23]. Men had high SBP (≥130 mmHg) (Table 1). However, women showed higher BMI, triglycerides, and total cholesterol levels than men (Table 1).

**Figure 2:**
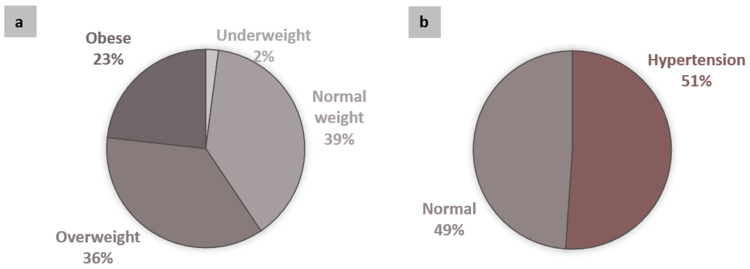
Distribution of nutritional status (**a**) and prevalence of hypertension (SBP ≥ 130 mm Hg and/or DBP ≥ 85 mm Hg) (**b**) in the participants (*n* = 818).

A high prevalence of overweight and obesity (BMI ≥ 25.0 kg/m^2^) and hypertension was found (Figure 2). 

**Figure 3:**
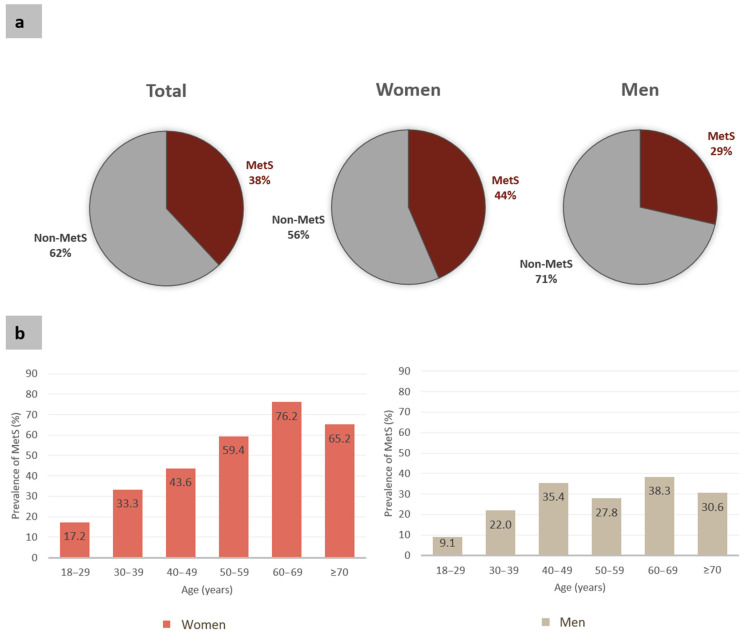
Prevalence of metabolic syndrome (MetS) for all participants, women and men (**a**) and according to the different age ranges. (**b**) Prevalence of MetS was higher among women (Fisher’s exact test, *p* < 0.0001).

A total of 311 individuals (38.0%; 95% CI 34.7–41.4) were diagnosed with MetS, with a significantly higher prevalence in 60–69 years-old individuals and among women (43.5%; 39.2–47.9) than men (28.6%; 23.5–34.0) (Figure 3).

No significant difference was detected in the ancestry profiles between the individuals with and without MetS (Table 2).

The relatively high number of participants in the Tapajós River basin and in the Tucuruí Lake region (Figure 1) allowed us to analyze possible differences between regions. A similar distribution of nutritional status was found between regions although hypertension prevalence was higher in Tucuruí (Supplementary Table S1 and Figure S1). The prevalence of isolated diastolic hypertension (IDH, defined as a DBP > 90 mmHg and an SBP < 140 mmHg) in Tucuruí and Tapajós was 7.7% and 1.4%, respectively. Interestingly, individuals with IDH in Tucuruí showed a mean age of 40.5 (±11.4) years old.

The MetS prevalence was similar in both regions, Tapajós (36.9%; 95% CI 32.3–41.8) and Tucuruí (39.1%; 34.3–44.1) (Fisher’s exact test, *p* > 0.05) (Supplementary Figure S2). Participants with MetS had significantly worse levels in all parameters as compared to individuals without MetS. Significant differences were found in individuals with MetS between the Tapajós and Tucuruí regions (Table 3). 

**Figure 4:**
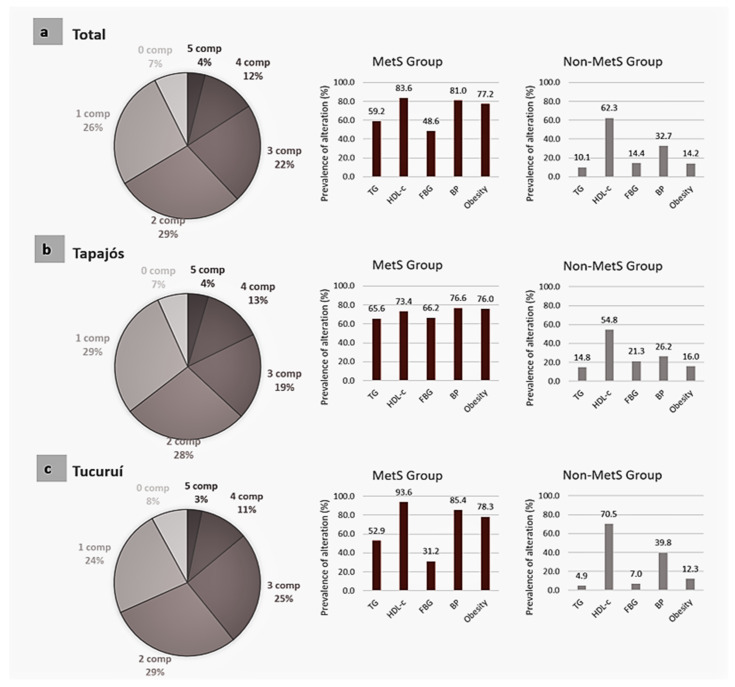
Analysis of metabolic syndrome (MetS) components (comp) for all participants (horizontal (**a**)), and those from Tapajós (horizontal (**b**)) and Tucuruí (horizontal (**c**)) regions. Pie charts show the participants’ distribution with 0 to 5 altered components. Bar graphics indicate the frequency of each altered component (high triglycerides, TG; low HDL cholesterol, HDL-c; high fasting blood glucose, FBG; high blood pressure, BP; and obesity-considering waist circumference, or BMI when waist circumference was not available) in participants with MetS (MetS Group) and without it (Non-MetS Group).

Figure 4 shows the distribution of participants according to the number of altered components and the frequency of each altered components of the MetS in individuals with and without the syndrome. Although 61.9% (95% CI 58.6–65.3) of the participants were not diagnosed with MetS, most of them already showed one or two altered components and only 7.3% (5.6–9.3) of the total population had none. No significant difference in this prevalence was seen in both locations (Figure 4, pie charts). Looking into the frequencies of the altered components (Figure 4, bar graphics), the three most frequent altered components in individuals with MetS were low HDL-c levels, followed by hypertension and obesity. 

Moreover, a low HDL-c level was also found in a large portion (as high as 70%) of the population without MetS, in both Tapajós and Tucuruí regions (Figure 4), meaning that over three of five individuals from Amazonian riverine populations without MetS already presented low HDL-c. Although the second most prevalent condition in those without MetS was hypertension, the profile in Tapajós and Tucuruí was slightly different, with similar prevalence to other components in Tapajós but a clear predominance of hypertension in Tucuruí as compared to other alterations. Additionally, the most frequent profiles of associated components were analyzed. In the 215 participants presenting only one altered MetS component, the low HDL-c was the most prevalent (found in 65.6% of these individuals). For individuals with two altered components, the most frequent combinations were low HDL-c and hypertension. For individuals with three altered components (and consequently with MetS diagnosis), the combination of hypertension, low HDL-c, and obesity was the most frequent found in 36% of these individuals.

## 4. Discussion

This study expands our state-of-science based on two main highlights: First, this is the first study investigating MetS in Amazonian riverine populations; and second, it is the largest epidemiological cohort (818 participants) ever evaluated using invasive sampling in non-urban populations of the Amazon. 

Interestingly, two recent systematic reviews demonstrated that epidemiological studies with riverine populations usually include 200 participants approximately to reach conclusions of epidemiologic value [24,25], supporting the representativity of our sample size. Moreover, studies on MetS prevalence in the Amazon are extremely scarce and usually limited to urban or indigenous populations [26,27,28,29,30]. Over 4 million people in the Brazilian Amazon are registered as rural population but this number includes indigenous people, *quilombolas* (African-descendants), people living at rural areas far from the rivers, and riverine populations [31]. Riverside communities are composed by non-indigenous populations of the Amazon, presenting a specific profile with an intimate relationship with the surrounding environment in which the river is central to the lifestyle [11,15,17,32]. These riverine communities are sometimes considered the “invisible” population of the Amazon because they do not have international or institutional visibility (different for indigenous groups or urban populations), and they hardly appear in the national statistics [17]. These two aspects are essential to understand the impact and the height of the conclusions on a population scarcely studied but of fundamental importance for the preservation of our environment, which shares features (low income, poor access to health services, traditional practices, etc.) with other vulnerable populations of the Southern Hemisphere. 

Large rivers and their tributaries comprise the central element in the traditional riverine lifestyle (the main route for population displacement; fish-based diet; fishery as the main source of income) [16,17]. This subsistence economy is mainly based on familiar agriculture (fruits, vegetables, manioc flour, etc.) and fishing [17]. Thus, the classical picture of the riverine lifestyle should be relatively healthier (surrounding by nature, less stressful life, fish as the main protein of the diet, etc.) than that of the urban dweller. 

Despite this traditional lifestyle, our preliminary results have shown a high prevalence of individuals at CVD risk according to anthropometric measures such as waist or neck circumferences [17]. Our large cohort here revealed median values of overweight and dyslipidemia, undesirable levels of total cholesterol, low HDL and/or high non-HDL cholesterol in both men and women (Table 1), confirming the increased risk for CVD in this population. The distribution of nutritional status showed a high prevalence of overweight and obesity (59%, Figure 2), higher than the two largest urban centers in Brazil, São Paulo (55.8%) and Rio de Janeiro (57.1%) [27]. Moreover, 23% of individuals were obese (with BMI values ≥ 30.0 kg/m^2^), a high prevalence as compared to São Paulo (19.9%) or Rio de Janeiro (21.7%) [27]. In fact, this obesity rate in Amazonian riverine populations was higher than the Brazilian national mean (20.3%) [27]. These data suggest that the global obesity epidemic may be established in these remote and traditional communities from the Amazon, since it was also described for the Amazonian indigenous population Xavante [33] and the afro-descendant “*quilombolas*” [34]. 

Another interesting aspect is the occurrence of atherogenic dyslipidemia in this population (Table 1). Surprisingly, as far as we know, this is the first study in the literature regarding the lipid profile of Amazonian riverine populations. Consequently, our data reveal an unprecedented and worrying scenario, which needs future studies to understand the mechanism underlying this atherogenic profile and its possible causes. 

One important factor associated with an atherogenic profile is hypertension. Our previous study had already indicated the high prevalence of hypertension in Amazonian riverine populations, including individuals with no history or previous diagnosis [15]. This was confirmed in the present study, analyzing a larger sample size: Although the median values for SBP and DBP were considered normal (Table 1), 51% of the total population had hypertension (Figure 2). This rate is significantly higher than that recently registered at the Brazilian Federal District-the highest hypertension prevalence among Brazilian states (28.5%) [27]. 

Dyslipidemia, obesity, hypertension, and high FBG are the main factors of MetS [2,35]. Therefore, we evaluated the prevalence of MetS in Amazonian riverine populations to identify those individuals who are at high risk of developing CVD and T2DM over the next 5 to 10 years [2]. MetS is not only a major public-health problem, but also a clinical one [2]. Thus, early MetS diagnosis is necessary to offer adequate management for an effective and accurate risk factor modification. Furthermore, the knowledge of the MetS epidemiological profile is the first step in public health to support the development of future strategies for reducing the prevalence and risk of both CVD and T2DM. 

This seminal study describes the MetS prevalence in a large cohort from Amazonian riverine populations concluding that 38% of the individuals had the syndrome (Figure 3). MetS prevalence worldwide range 2% to 66.9%, varying according to age, gender, race/ethnicity, and the criteria used for the diagnosis [35]. In Brazil, the weighted mean for the general prevalence of MetS is 28.9%, using the criteria of the American Heart Association [36]. However, our data show that the MetS prevalence in the Amazonian riverine population is higher than this value and that of urban populations in Brazil [36].

Moreover, taking medication for hypertension, hypertriglyceridemia, hypocholesterolemia-HDL, or diabetes is included as a criterion for MetS diagnosis [21], which increases the MetS prevalence compared to diagnosis based only on quantitative data from components. Consequently, the scenario in Amazonian riverine populations could be even worse than we reported. However, a conservative approach disregarding these drug treatments was performed here, considering that the compliance and continuity of chronic treatments are huge challenges in these communities (participants with a diagnosis of hypertension and/or diabetes frequently reported that they were not taking drugs because they had no money to buy them).

Also, we adopted the American Heart Association definition of MetS because it is more inclusive as compared to the International Diabetes Federation (IDF) and WHO definitions, which consider a pre-requisite [21]. MetS diagnosis by the American Heart Association definition just demands, at the least, any three of the possible altered components (see Section 2.3 of Materials and Methods), increasing the sensitivity of the diagnosis. Still, the application of the ethnic-specific cut-offs suggested by IDF for waist circumference (i.e., ≥90 cm and ≥80 cm for South Americans men and women, respectively) instead of the AHA definition (>102 cm for men and >88 cm for women) would eventually contribute to increase the MetS prevalence that we found here. 

MetS has been frequently related to the urban lifestyle, especially because urbanization is usually associated with an increased prevalence of hypertension, obesity, and dyslipidemia linked to high energy intake and sedentary life habits [37]. Thus, the high prevalence of MetS found in Amazonian riverine populations does not seem to be compatible with the riverine traditional lifestyle, which includes physical activity and diet based on local food production from horticulture, fishing, and foraging activities [38,39]. Indeed, forager–horticulturalist, pastoralist, and traditional farming populations are known to have a high physical activity ratio (calculated as the daily total energy expenditure of an individual divided by the daily basal metabolic rate) [40], strongly suggesting that changes in the nutrition or lifestyle could be partially responsible. 

A certain degree of nutrition transition has been shown in traditional Amazonian populations, such as indigenous [26,33] and riverine communities from the Tapajós basin [17,32,41,42,43]. In many rural areas, there was a shift in the subsistence-based economy to wage labor and, in many cases, to the Brazilian conditional cash transfer program, called “Bolsa Família” [17,32,41,43]. The money gave people an opportunity to obtain industrialized food, increasing the intake of these types of food and decreasing the traditional Amazonian food intake [17,42]. However, this phenomenon has not been detected in the Tucuruí region [17], and different factors could be contributing. 

Due to the high number of participants included in our study as compared to epidemiological studies in the Amazon, it was possible to perform an additional analysis according to each region to detect possible differences. The final number of participants included in each region was about twice the sample size frequently used to reach epidemiological conclusions with Amazonian riverine populations [24,25]. The participants from the Tucuruí region presented a worse profile than those from the Tapajós River basin, with significantly lower median values of HDL-c, and higher median values of SBP and DBP, total cholesterol, and non-HDL-c (Supplementary Table S1). In agreement with the worse atherogenic profile, the hypertension prevalence in Tucuruí was significantly higher than in the Tapajós basin (Supplementary Figure S1). Factors including the presence of environmental pollutants could be playing a role in cardiovascular risk. Both regions, Tapajós and Tucuruí, are knowingly affected by anthropogenic mercury contamination [11,14,15,44,45]. Therefore, in addition to possible genetic contributors (especially for low HDL-c levels), other factors that we have extensively documented, such as environmental contamination [11,14,15,16,43,45], could be influencing the progression of the syndrome. 

The mercury content in fish may be an important characteristic in this scenario, since fish remains, to date, the most consumed source of proteins in the diet of the Amazonian population (even in those regions where the phenomenon of nutrition transition has been detected) [16,17,42]. Dietary fish is a rich source of omega-3 poly-unsaturated fatty acids with a protective effect against cardiovascular events, particularly by reducing atherogenic lipid levels and increasing HDL-c levels [46,47,48]. Interestingly, fresh fish intake, as consumed by the Amazonian riverine population [17], had a more pronounced effect on modifying lipid levels as compared to omega-3 fatty acid supplements [46]. However, the high mercury content in fish in these regions [49] could be counteracting the beneficial role exerted by the fish-based diet. Mercury intoxication has been shown to have cardiovascular effects [50]. Two recent systematic reviews that performed dose–response metanalyses have concluded that there are non-linear associations between hair mercury and cardiovascular outcomes, including hypertension [51,52]. A significant correlation with hair mercury content and hypertension was already described in inhabitants from the Tapajós basin [53]. Interestingly, the riverine population from the Tucuruí region currently shows significantly higher mercury content in hair than that of the Tapajós basin [11], eventually contributing to the higher prevalence of hypertension in this region (Supplementary Figure S1). Moreover, although the consumption of Amazonian fruits could protect against the mercury deleterious effects [54], dietary methylmercury can also cause xenobiotic-mediated dysbiosis of the gut microbiome [55], that could eventually contribute to obesity in these individuals [56]. Further studies are necessary to understand the exact contribution of this pollutant in this scenario and other possible causes of this phenomenon. 

Similar to most of the studies on MetS prevalence in Brazilian adults [36], there was higher participation of women as compared to men in this study (Table 1). This also confirms previous data about participation according to gender in studies with Amazonian populations [57,58,59], and it has been already attributed to the fact that women may be more careful with health than men [59]. Still, women had a significantly higher prevalence of MetS (Figure 3), a trend also observed for each region separately (Supplementary Figure S2), and in Brazilian cities [36]. Indeed, the MetS prevalence was higher for women in all age ranges (Figure 3), even when analyzed separately for each region (Supplementary Figure S2), supporting the female gender as a possible additional risk factor for MetS in Amazonian riverine populations. Additional studies are necessary to analyze whether possible differences in physical activities between genders could also partially explain the different prevalence. 

There is a general agreement that MetS rates increase with each decade of life [2,60]. The distribution of women with MetS according to age showed a profile with the highest prevalence in the last decades of life, but the prevalence in men has a more homogeneous pattern with a peak at 60–69 years old (Figure 3). These data are similar to those of other populations with the highest prevalence of MetS in older subjects, pointing to aging as an important risk factor [29,61]. Interestingly, there was a decrease in the prevalence in aged people (≥70 years old) (Figure 3). This could be an artefact of the small sample size of this group or could even be related to death at this age—although we are not able to confirm this due to the absence of accurate demographic data for these populations. However, further studies need to be done to better understand this process. Interestingly, the MetS prevalence in young adults (18–30 years) was 15.4% (17.2% for women and 9.1% for men), three times higher than the overall prevalence (4.8%) found in a pooled analysis with studies of several countries [60]. Because MetS and its components represent a lifetime of increased CVD risk [60], our data support the need for starting intervention in these riverine populations as early as the thirties for both women and men, considering the curves of MetS prevalence according to age. 

According to AHA [21], the diagnosis of MetS is based on the presence of at least three of five possible altered components (hypertension, obesity, high triglycerides, low HDL-c, and/or high FBG). The presence of more altered components reveals a worse situation for the individuals with MetS, and even the analysis of the different MetS components in individuals without MetS may provide important information to develop prevention strategies. Thus, to better understand this phenomenon in these Amazonian riverine populations, the median values of the MetS components in each region and the prevalence of each altered component were analyzed (Table 3 and Figure 4).

As expected, all the median values of the MetS components for the group of participants diagnosed with MetS were outside healthy limits, except for FBG in the Tucuruí region (Table 3). Of note, the different MetS profiles of the individuals with MetS in Tucurí is characterized by significant differences in median values of blood pressure and HDL-c levels, pointing to these two factors being particularly problematic in this region. Median DBP was especially high, suggesting worse diastolic hypertension than in Tapajós. Indeed, the IDH prevalence was higher in Tucuruí than in Tapajós. An increased risk of cardiovascular events is associated with IDH, in particular among young hypertensive patients (<50 years old) [62]. Although IDH is a largely underestimated risk factor for CVD mortality [63], it was the most frequent form of diastolic hypertension in young adults (<40 years old) in the NHANES cohort [64]. Accordingly, our results revealed a relatively low mean age in individuals with IDH in Tucuruí, for the first time identifying a proportion of a relatively young population at high risk in these Amazonian riverine populations. Careful monitoring over the time of individuals with IDH in the short- or middle-term is recommended, treating not only hypertension but all cardiovascular risk factors [62,63]. Our results claim attention to the importance of epidemiologic knowledge for developing tailored prevention strategies that increase the efficacy and the impact of the public health programs in vulnerable populations. 

Moreover, the median values of HDL-c were generally below the limit (Table 1 and Table 2) and participants with MetS in Tucuruí had a significantly lower median. Genetic susceptibility and disorders, as well as secondary factors such as smoking, T2DM, MetS, and abdominal obesity, are known determinants of low HDL-c [65]. The prevalence of MetS and obesity was similar in both locations (Supplementary Figures S1 and S2), so these factors do not seem to be the main responsible for HDL-c levels difference seen between the two regions (Table 3). In addition, T2DM does not seem to have an important role in this case, since the glucose level is significantly higher in Tapajós than Tucuruí (Table 3). Finally, Brazilians in general do not smoke much (as compared with European or Northern American populations) [66], and the low income of the Amazonian riverine population is unlikely to afford this addiction (personal observations). Especially in Tucuruí, access is very difficult due to the isolation and remote setting of this region, suggesting that smoking is not a contributor to these differences in individuals with MetS. The average serum HDL-c levels below the normal limit or very close to it in these remote areas may point to the genetic background as the main cause.

Although the lack of information on lifestyle factors (smoking, sedentarism, etc.) represents a limitation of the present work, as the first approach on MetS in Amazonian riverine populations, the objective of this study was merely descriptive. Based on the alarming results described here, it is imperative that additional studies investigate potential causes related to lifestyle as well as other factors including but not limited to pollutants exposure and genetic background.

To better understand the MetS phenomenon in this vulnerable population, we also analyzed the prevalence of each altered component in both individuals with and without MetS in both regions (Figure 4). Although there was a high prevalence of people with MetS, only 16% of all participants had four or five altered components (Figure 4, pie charts), with most individuals of the MetS group showing only three altered components (the minimum for MetS diagnosis), indicating the possibility of reversion of this high prevalence after a targeted and effective intervention. Furthermore, the analysis of individuals without MetS showed that approximately 30% of the population already has two altered components (Figure 4, pie charts), indicating that in the case of no intervention in the near future, the MetS prevalence may rise dramatically up to 70% (38% already diagnosed plus 30% with two altered components) implying a huge impact in the public health system. Moreover, this distribution was quite similar in Tapajós and Tucuruí supporting that this would be a general distribution of prevalence in the Amazonian riverine population.

The most frequent altered component of the MetS criteria in all participants was the low HDL-c level (Figure 4). Interestingly, the HDL-c level was also below the limit in a large portion of participants without MetS, independently of the region (Figure 4). This is also confirmed with the most frequent profiles found in individuals with only one altered component of the MetS, where low HDL-c was the most prevalent (65.6%), followed by hypertension (20%). Indeed, 576 individuals of 818 in this study had a low HDL-c level (70.4%), which is surprisingly high as compared with frequencies described by the National Health and Nutrition Examination Survey in adults of United States (31% and 12% for men and women, respectively) [67]. As highlighted before, a strong inherited basis is linked to blood levels of HDL-c, with heritability estimates of 40–60% [68]. A number of genes has been already identified as playing a role in lowering HDL-c levels, such as ABCA1, APOA1, APOC3, APOA5, and APOE [68]. Unfortunately, epidemiological data on the genetic background of the Amazonian population are extremely scarce. Two recent reports have demonstrated the high prevalence of APOE4 allele in Amazonian riverine populations that would be associated with the indigenous ancestry [11,15]. Both indigenous ancestry and the APOE4 allele have already been associated with a significant decrease in plasma HDL-c levels and a higher prevalence of CVD [69,70]. Due to the high proportion of indigenous ancestry (approximately one-third as revealed by our results, Table 2), it could contribute, at least in part, to this widespread alteration observed in this population. HDL-c is a significant predictor of major cardiovascular events and its inverse relationship with them is one of the most robust epidemiological observations ever made [71]. Low HDL-c level remains a key element on the prevention of CVDs in the main treatment guidelines [72] and our data demonstrate that adequate management of this alteration would be essential for effective prevention strategies in public health programs in the Amazon.

**Figure 5:**
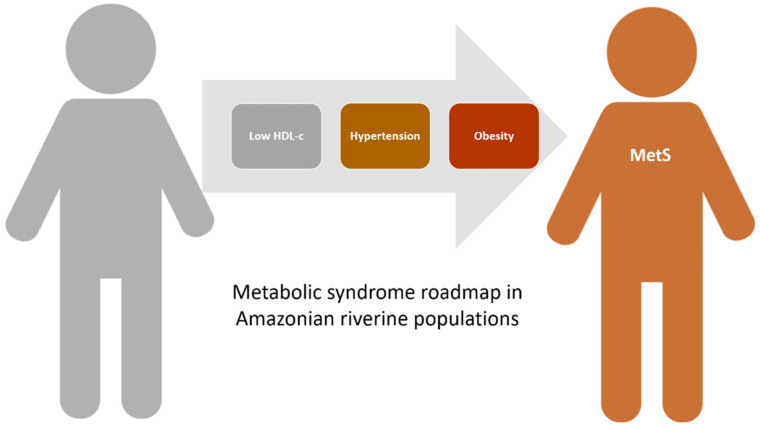
Roadmap leading to metabolic syndrome (MetS) in Amazonian riverine populations based on the prevalence found in this study.

The second most prevalent MetS component was hypertension, followed by obesity (Figure 4). This was also confirmed by the most common associations (low HDL-c plus hypertension and low HDL-c plus hypertension plus obesity) in individuals with two and three altered components, respectively. Thus, based on the results of this study we can figure a likely sequence of changes that would add up in the temporal progression leading to MetS in Amazonian riverine populations (Figure 5). 

## 5. Conclusions

MetS is a multifactorial disorder, therefore knowing the factors that contribute to its outcome is fundamental, particularly when possible environmental and genetic factors could be associated with it. Based on our data, we can tentatively propose a roadmap to MetS progression in Amazonian riverine populations based on the most prevalent MetS criteria and profiles. Initially, individuals would present low HDL-c levels, later progressing to elevated blood pressure characterizing hypertension, and ultimately reaching MetS with obesity (Figure 5). Knowing this roadmap can be very useful for the design of preventive strategies to halt the progression and establishment of the syndrome and reduce the CVD risk in Amazonian riverine populations. Our data reveal for the first time that only 7–8% of adults had no cardio-metabolic abnormalities related to MetS, highlighting the urgent need for the development of tailored interventions to urgently reduce the high CVD risk found in Amazon. Additionally, this study opens new questions including the possible genetic contribution lowering HDL-c levels or the exact contribution of mercury exposure to hypertension in Amazonian inhabitants that must to be better investigated to understand the mechanisms underlying this alarming scenario detected in the Amazon. 

## Figures and Tables

**Table 1:** Anthropometric, clinical, and biochemical profiles of the participants. Data are presented as median and interquartile ranges. Median values out of the acceptable range recommended by the Brazilian Guidelines are highlighted in bold.

	Total *n* = 818 (100%)	Women *n* = 517 (63.2%)	Men *n* = 301 (36.8%)	Women vs. Men *p*-Value *
Anthropometric and clinical profile				
Age, year	47 (33–57)	44 (31–55)	51 (40–60)	<0.0001
Height, cm	155 (151–163)	152 (149–157)	164 (159–169)	<0.0001
Weight, kg	64.5 (56.4–74.4)	61.9 (54.5–71.3)	68.6 (60.2–78.0)	<0.0001
BMI, kg/m^2^	**25.7 (23.1–29.5)**	**26.8 (23.4–30.4)**	**25.5 (23.3–28.4)**	0.0003
Waist circumference, cm	90 (82- 98)	89 (82–98)	90 (83–98)	0.3544
Systolic BP, mmHg	126 (114–140)	122 (110–138)	**130 (120–144)**	<0.0001
Diastolic BP, mmHg	80 (71–89)	79 (70–87)	81 (74–91)	0.0002
Biochemical profile				
Fasting blood sugar, mg/dL	90 (80–101)	90 (80–104)	90 (81–99)	0.6943
Serum triglyceride, mg/dL	119 (80–158)	123 (86–160)	107 (71–153)	0.0050
Serum total cholesterol, mg/dL	**195 (163–226)**	**199 (165–233)**	**192 (161–222)**	0.0272
Serum HDL cholesterol, mg/dL	**39 (32–47)**	**39 (33–48)**	**38 (31–45)**	0.0053
Serum non-HDL cholesterol, mg/dL	**153 (122–187)**	**155 (122–190)**	**151(121–181)**	0.2350

*: Mann-Whitney test; BMI, body mass index; BP, blood pressure; HDL, high density lipoprotein.

**Table 2:** Ancestry profile of Amazonian riverine individuals participating in this study, diagnosed with metabolic syndrome (MetS) and without it (non-MetS). Data are presented as median and interquartile ranges.

Ancestry	Total ^a^ *n* = 728	MetS *n* = 281	Non-MetS *n* = 447	*p*-Value ^b^
European, %	42.1 (32.2–52.3)	41.6 (30.5–50.2)	42.3 (32.7–51.4)	0.2608
Amerindian, %	31.9 (23.0–42.2)	32.3 (23.3–44.6)	31.8 (22.6–41.3)	0.0856
African, %	22.9 (16.4–30.6)	22.5 (15.7–29.9)	23.0 (17.0–31.5)	0.2914

^a^: Data of ancestry were available for 728 participants of this study. ^b^: Mann–Whitney test.

**Table 3:** Sex distribution and median values (and interquartile ranges) of the metabolic syndrome (MetS) components in individuals with and without the syndrome and *p*-values of differences between individuals with and without MetS in each region and for individuals with MetS between the two regions, the Tapajós River basin and the Tucuruí Lake. Median values out of the limits recommended by the Brazilian Guidelines are highlighted in bold.

	Tapajós Area (*n* = 417)			Tucuruí Area (*n* = 401)			MetS
	MetS (*n* = 154)	non-MetS (*n* = 263)	MetS vs. Non-MetS	MetS (*n* = 157)	non-MetS (*n* = 244)	MetS vs. Non-MetS	Tapajós vs. Tucuruí
Sex							
Women	118 (76.6%)	159 (60.5%)	0.0008 ^a^	107 (68.2%)	133 (54.5%)	0.0068 ^a^	0.1009 ^a^
Men	36 (23.4%)	104 (39.5%)		50 (31.8%)	111 (45.5%)		
MetS components							
BMI, kg/m^2^	**29.5 (26.9–33.0)**	24.3 (21.8–27.1)	<0.0001 ^b^	**30.3 (26.9–32.9)**	24.03 (21.9–27.0)	<0.0001 ^b^	0.5976 ^b^
Waist circumference, cm	98 (91–103)	86 (79–94)	<0.0001 ^b^	97 (92–105)	85 (79–94)	<0.0001 ^b^	0.6670 ^b^
Systolic BP, mm Hg	**136 (129–149)**	116 (107–126)	<0.0001 ^b^	**140 (131–153)**	121 (111–129)	<0.0001 ^b^	0.0532 ^b^
Diastolic BP, mm Hg	81 (74–90)	76 (68–81)	<0.0001 ^b^	**91 (82–98)**	74 (66–84)	<0.0001 ^b^	<0.0001 ^b^
Fasting blood sugar, mg/dL	**109 (94–127)**	88 (79–97)	<0.0001 ^b^	93 (84–103)	84 (78–92)	<0.0001 ^b^	<0.0001 ^b^
Serum triglyceride, mg/dL	**166 (129–195)**	114 (79–138)	<0.0001 ^b^	**155 (112–193)**	86 (60–108)	<0.0001 ^b^	0.0443 ^b^
Serum HDL-c, mg/dL	**38 (33–49)**	42 (34–54)	0.0466 ^b^	**33 (28–39)**	40 (33–46)	<0.0001 ^b^	<0.0001 ^b^

Abbreviations: BMI, body mass index (calculated as weight in kilograms divided by the square of height in meters); BP, blood pressure; HDL-c, high density lipoprotein cholesterol. ^a^: Fisher’s exact test; ^b^: Mann-Whitney test.

## Data Availability

All data of this study can be requested to the corresponding author (ecrespo@ufpa.br or maria.elena.crespo.lopez@gmail.com).

## Supplementary Materials

The following are available online at https://www.mdpi.com/article/10.3390/jcm10163630/s1, Figure S1: Distribution of nutritional status and prevalence of hypertension in Tapajós and Tucuruí, Figure S2: Prevalence of MetS in all participants, women and men, and according to age ranges of each sex in Tapajos and Tucuruí regions, Table S1: Anthropometric, clinical and biochemical characteristics of participants of the Tapajós River basin and the Tucuruí Lake.
