# Supplementary material for: Living in the Southern Hemisphere: Metabolic Syndrome and Its Components in Amazonian Riverine Populations
Source: J Clin Med. 2021 Aug 17;10(16):3630. doi: 10.3390/jcm10163630 (PMC8396977; doi:10.3390/jcm10163630)
Supplement: Supplementary file 1 [file jcm-10-03630-s001.zip › jcm-1261225-supplementary.pdf]

SUPPLEMENTARY MATERIAL

Living in the Southern Hemisphere: metabolic syndrome and its components in Amazonian riverine populations

Supplementary Table S1…………………………………page 2

Supplementary Figure S1………………………………..page 3

Supplementary Figure S2………………………………..page 4

Supplementary Table S1. Anthropometric, clinical and biochemical characteristics of participants of the Tapajós River basin and the Tucuruí Lake. Data are presented as frequency or median (and interquartile ranges). Median values out of the limits recommended by the Brazilian Guidelines are highlighted in bold red.

	**Tapajós** *n* **= 417**	**Tucuruí** *n* **= 401**	Tapajós *vs* Tucuruí *p*-value
Sex			
Women, *n* (%)	66.4	59.9	0.0593[a]
Men, *n* (%)	33.6	40.1	
Anthropometric and clinical profile			
Age, y	47 (33–59)	46 (34–56)	0.3406[b]
Height, cm	155 (150–162)	156 (151–163)	0.1097[b]
Weight, kg	63.8 (55.7–74.0)	65.4 (57.2–75.2)	0.1685[b]
BMI, kg/m^2	**26.0 (23.3–29.4)**	**26.2 (23.4–29.9)**	0.5519[b]
Waist circumference, cm	91 (82.5–99.0)	89 (82–98)	0.2052[b]
Systolic BP, mmHg	122 (110–138)	129 (117–145)	<0.0001[b]
Diastolic BP, mmHg	78 (70–85)	82 (72–92)	<0.0001[b]
Biochemical profile			
Fasting blood sugar, mg/dL	93 (83–110)	87 (79–97)	<0.0001[b]
Serum triglyceride, mg/dL	128 (91–166)	103 (68–146)	<0.0001[b]
Serum total cholesterol, mg/dL	**190 (158–229)**	**199 (171–225)**	0.0165[b]
Serum HDL cholesterol, mg/dL	**40 (33–53)**	**37 (31–44)**	<0.0001[b]
Serum non-HDL cholesterol, mg/dL	**146 (114–186)**	**162 (132–187)**	0.0002[b]

Abbreviations: BMI, body mass index; BP, blood pressure; HDL, high density lipoprotein. [a] Fisher's exact test and Mann–Whitney test to compare percentages and median values, respectively.

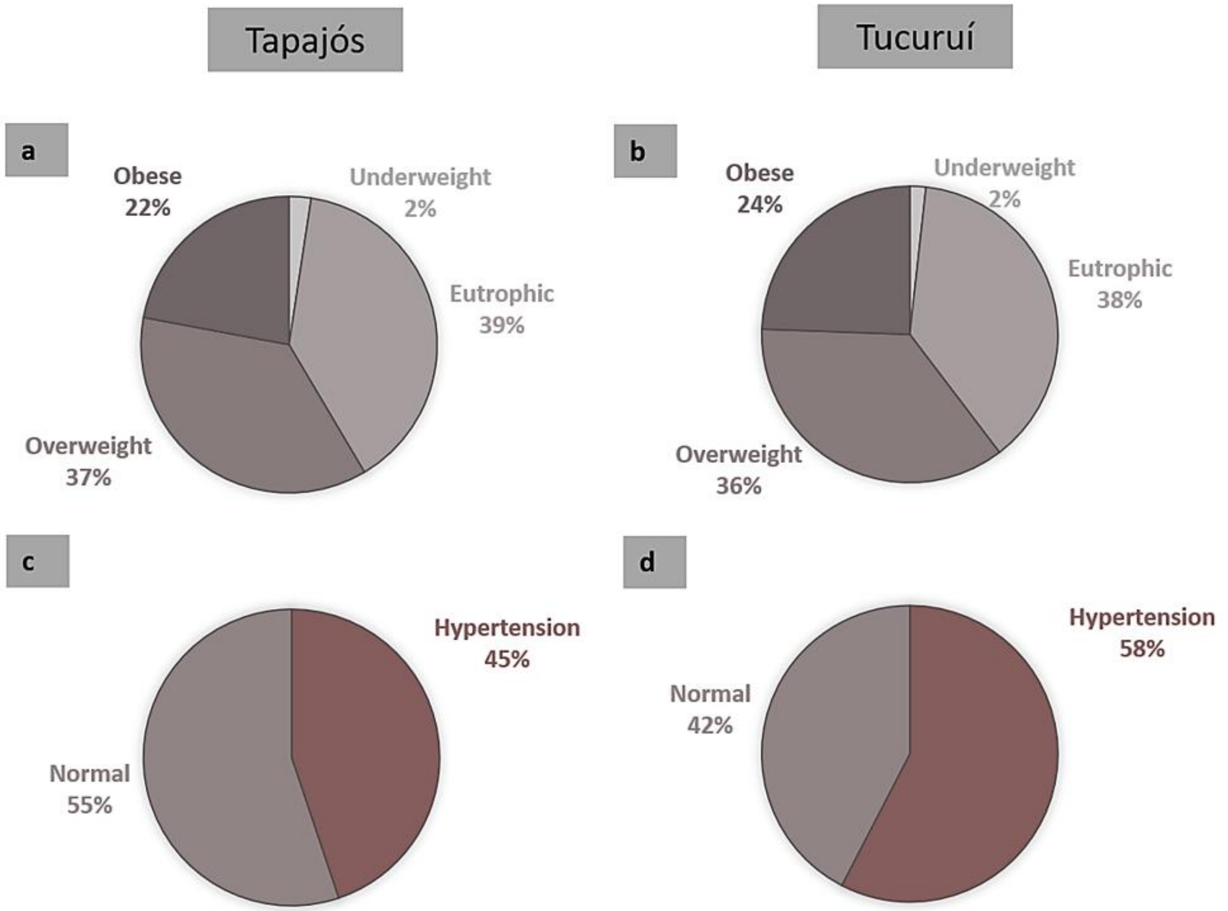

Supplementary Figure S1. Distribution of nutritional status and prevalence of hypertension in Tapajós (**A** and **C**, respectively) and Tucuruí (**B** and **D**, respectively).

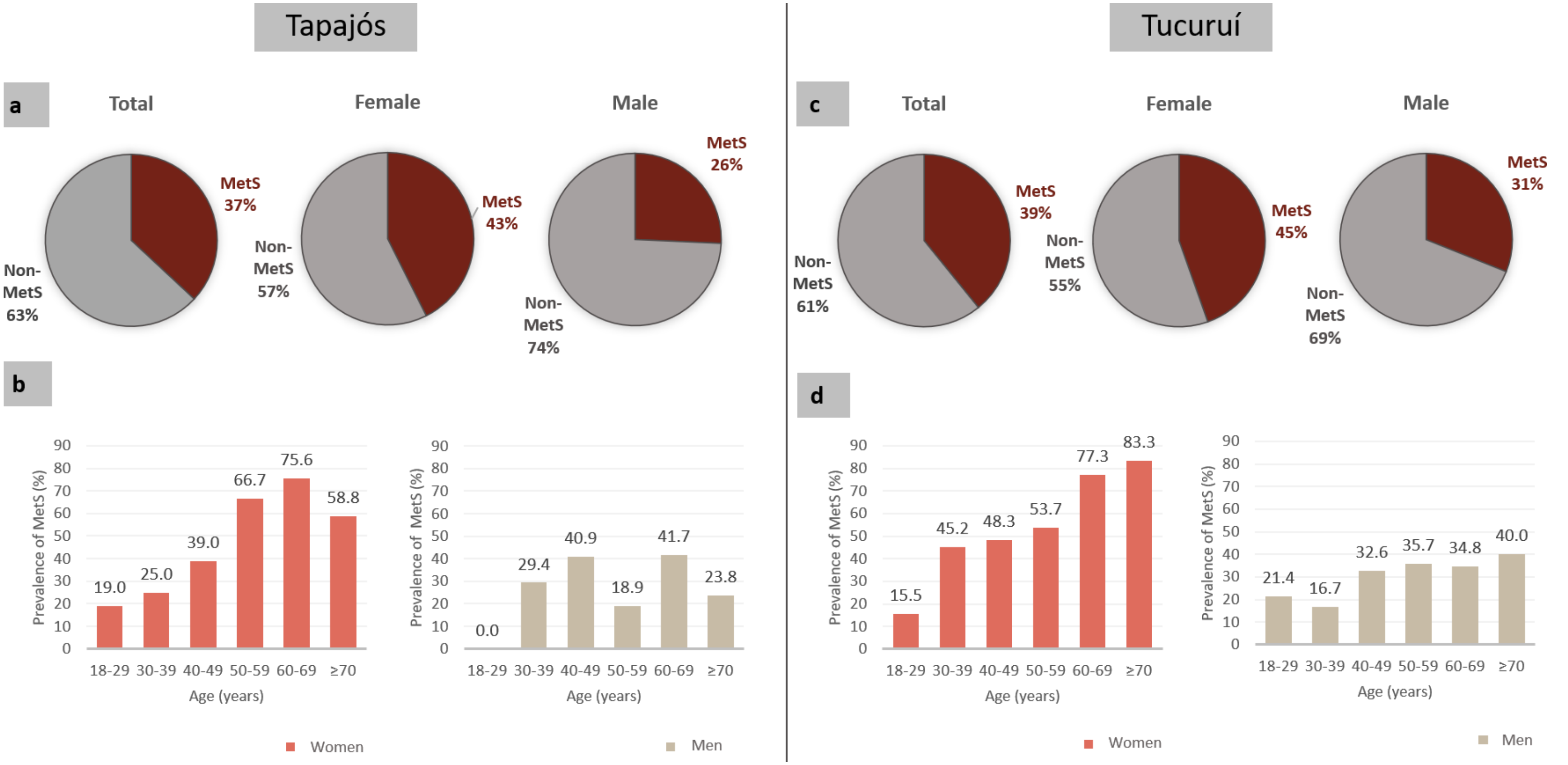

Supplementary Figure S2. Prevalence of MetS in all participants, women and men (pie charts), and according to age ranges of each sex (bar graphics) in Tapajos (**A** and **B**) and Tucuruí (**C** and **D**) regions.
